# Role of cathepsin K in the expression of mechanical hypersensitivity following intra-plantar inflammation

**DOI:** 10.1038/s41598-022-11043-9

**Published:** 2022-05-02

**Authors:** Maha Paracha, Amit Thakar, Rebecca A. Darling, Shaun S. Wulff, Dan C. Rule, Sreejayan Nair, Travis E. Brown

**Affiliations:** 1 Animal Sciences Graduate Program, University of Wyoming, Laramie, WY 82071 USA; 2 Neuroscience Graduate Program, University of Wyoming, Laramie, WY 82071 USA; 3 School of Pharmacy, University of Wyoming, Laramie, WY 82071 USA; 4 Department of Integrative Physiology and Neuroscience, Washington State University, Pullman, WA 99164 USA; 5 Department of Mathematics and Statistics, University of Wyoming, Laramie, WY 82071 USA

**Keywords:** Chronic pain, Chronic pain

## Abstract

Persistent/chronic inflammatory pain involves multiple pathophysiological mechanisms and is far more complex than acute/momentary pain. Current therapeutics for chronic inflammatory pain are often not effective because the etiology responsible for the pain is not addressed by traditional pharmacological treatments. Cathepsin K is a cysteine protease that has mostly been studied in the context of bone and joint disorders. Previous work by others has shown that inhibition of cathepsin K activity reduces osteoarthritis-associated nociception in joints. However, the role of cathepsin K in cutaneous inflammation is understudied. We assessed the effectiveness of genetic deletion or pharmacological inhibition of cathepsin K in male mice on the expression of nocifensive behaviors after formalin injection or mechanical and thermal hypersensitivity after injection of complete Freund’s adjuvant (CFA) into the mouse hind paw. Our data demonstrate that cathepsin K knockout mice (*Ctsk*^−/−^) have a reduction in nocifensive behaviors in the formalin test. In addition, *Ctsk*^−/−^ do not develop mechanical hypersensitivity after CFA injection for up to 7 days. Moreover, we found that inhibition of cathepsin K reduced mechanical hypersensitivity after CFA injection and mRNA levels, protein levels, and cathepsin K activity levels were elevated after CFA injection. Based upon our data, cathepsin K is indicated to play a role in the expression of chemically-induced cutaneous hypersensitivity, as *Ctsk*^−/−^ mice do not develop mechanical hypersensitivity and show a reduction in nocifensive behaviors. Further research is needed to determine whether attenuating cathepsin K activity may generate a clinically relevant therapeutic.

## Introduction

Pain conveys information about our bodies when something may be awry, signals us when we need to protect body parts from further damage, and helps us make associations between stimuli that may be harmful and threatening to us^1^. The “problem” with pain arises when acute/momentary pain becomes persistent/chronic due to maladaptations within the peripheral and/or central nervous systems^2^. Inflammation can play a critical role in the transition of acute to chronic pain, but our understanding of the underlying mechanisms is incomplete, and currently available analgesics do not address the underlying etiology^2–8^. Of particular interest to our group is the role of proteases that either directly or indirectly work in concert with inflammation to bring about these changes.

Cathepsins are endogenous proteases mostly found within lysosomes^9,10^. While cathepsins are important in intracellular protein degradation, energy metabolism, and immune responses, they are also implicated in wide range of diseases including cancer, cardiovascular diseases, and inflammatory neurological diseases^11^. When secreted extracellularly under physiological conditions, cathepsins degrade protein components of the extracellular matrix^12^. In disease states, the role of cathepsins goes beyond simple proteolysis, as cathepsins participate in cytokine and chemokine processing during inflammation and mediate pain signaling^13–17^. Specifically, cathepsin S facilitates both inflammatory pain^18^ and neuropathic pain^19,20^. After nerve injury, cathepsin S takes part in the transition process from acute to chronic pain^21^. It exerts its effect by activating the protease-activated receptor (PAR) 2^18,22^. Similarly, it has been shown that cathepsin B promotes chronic inflammatory pain after peripheral inflammation^23^, while cathepsin E (an aspartic proteinase^24^) contributes to the generation of neuropathic pain in autoimmune encephalitis^25^, and cathepsin G (a serine proteinase^26^) plays a role in the development of chronic post-surgical pain^27^. Therefore, a prominent role for cathepsins in various pain paradigms is beginning to emerge.

Traditionally, cathepsin K has been studied in the context of bone and joint disorders^15,28,29^. However, there is growing evidence that cathepsin K has a critical role in chronic inflammatory pain signaling. For instance, in a guinea pig model for osteoarthritis in knees, prolonged cathepsin K inhibition reduced joint nociception^30^. Furthermore, inhibition of cathepsin K activity has been shown to have analgesic effects in a model of osteoarthritis^30^. We wanted to expand upon these studies and determine if cathepsin K has a role in models of cutaneous inflammation. To determine this, we assessed the effectiveness of genetic deletion or pharmacological inhibition of cathepsin K activity in male mice on the expression of nocifensive behaviors after formalin injection or mechanical and thermal hypersensitivity after injection of complete Freund’s adjuvant (CFA) into the mouse hind paw. Our data suggest that cathepsin K plays a role in the expression of inflammatory-induced hypersensitivity as cathepsin K knockout mice (*Ctsk*^*−/−*^) do not develop mechanical hypersensitivity and have a reduction in nocifensive behaviors. In addition, our data suggests that acute local pharmacological inhibition, unlike in models of osteoarthritis, produces an analgesic effect resulting in the attenuation of hypersensitivity after CFA injection. Furthermore, we observed increases in cathepsin K mRNA, protein, and activity after CFA exposure. Taken together, our data indicates cathepsin K is elevated after CFA injection and contributes to CFA-induced mechanical hypersensitivity. Further research is needed to determine the parameters by which inhibition of cathepsin K activity produces analgesia and whether attenuating cathepsin K activity may generate a clinically relevant therapeutic.

## Results

### Cathepsin K knockout (***Ctsk***^−/−^) mice do not develop hypersensitivity

Two chemically induced inflammatory models of pain were assessed to determine whether cathepsin K is necessary for the expression of inflammatory pain. The first model injected formalin into the hind paw. Formalin is the aqueous solution of 37% formaldehyde and is a chemical irritant^31^. In the formalin test, dilute formalin is injected subcutaneously into the hind paw. This test mimics a continuous chemical-induced tissue injury^31^. This causes the animal to display unprovoked nocifensive behaviors^32^. This display of nocifensive behaviors can be seen in two phases, the early phase, and the late phase. The response seen in the early phase is caused mostly by the activation of C-fibers. In the late phase, pain is thought to be produced by a combined effect of the inflammatory pain mediators released from the inflamed tissue and the functional changes in the dorsal horn^31^.

**Figure 1:**
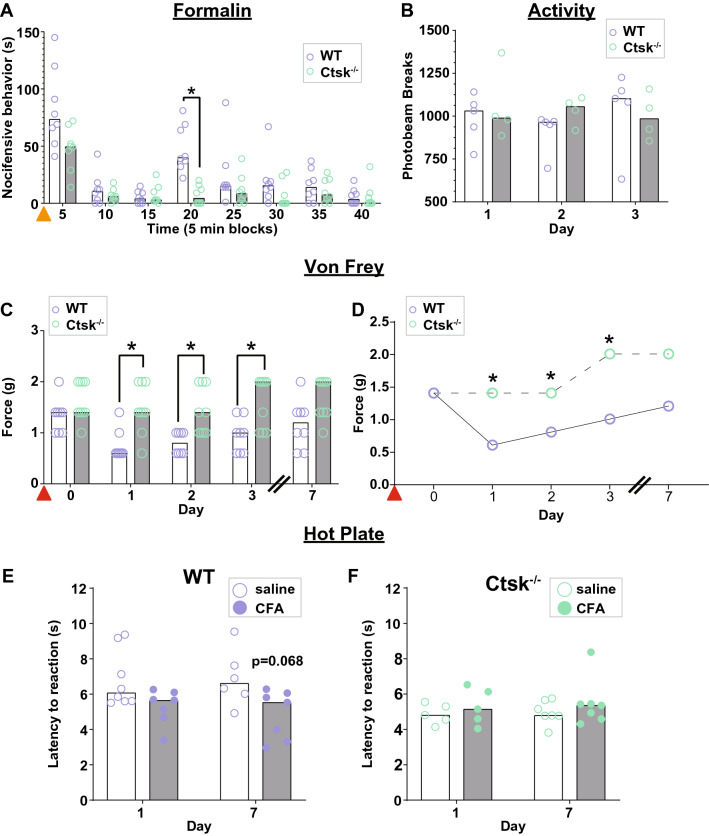
Cathepsin K knockout mice do not develop hypersensitivity to mechanical or thermal stimuli and have an attenuation in chemically induced nocifensive behaviors. (**A**) Formalin test, cathepsin K knockout (*Ctsk*^−/−^) mice have a reduction in nocifensive behaviors associated with the longer-lasting, more persistent phase of the formalin test compared to wild type (WT) mice. Orange triangle indicates the time of formalin injection. (**B**) General locomotor activity between WT and *Ctsk*^−/−^ mice. (**C**,**D**) Compared to the WT mice, *Ctsk*^−/−^ mice do not show mechanical hypersensitivity after complete Freund’s adjuvant (CFA) injection, shown as scatter plot overlayed with a bar graph representing the median; (**D**) line graph of the same data. Red triangle indicates the time of CFA injection. (**E**) CFA-injected WT mice show a trend towards thermal hypersensitivity after CFA injection, which is not seen in the CFA-injected *Ctsk*^−/−^ mice (**F**). Data are represented at scatter plots of individual mice with bar graphs overlayed indicating the median value. *p < 0.05 multiple Mann–Whitney test between WT and *Ctsk*^−/−^ mice for each time point.

The formalin-evoked nocifensive response was compared amongst wild type (WT) and *Ctsk*^−/−^mice. *Ctsk*^−/−^ mice exhibited a significant reduction in nocifensive behaviors compared to the WT mice 15–20-min (late phase) after formalin exposure (Fig. 1A; WT = 40 s, n = 8, *Ctsk*^*−/−*^ = 4.50 s, n = 8; Mann–Whitney U = 0.0, p = 0.001) and a trend towards an attenuation in nocifensive behaviors in the early phase (0–5 min) in *Ctsk*^−/−^ compared to WT mice (WT = 73.5 s, n = 8, *Ctsk*^*−/−*^ = 49.5 s, n = 8; Mann–Whitney U = 12.0, p = 0.12), indicating that cathepsin K might be necessary for the expression of nocifensive behaviors during the late phase of the formalin test. Both *Ctsk*^−/−^ and WT mice had similar paw diameters and weights after formalin injection, indicating both groups developed inflammatory edema and swelling (data not shown). To rule out that the reduction in nocifensive behaviors could not be attributed to a reduction in locomotor activity, general locomotor activity between the two groups was assessed.

No difference in basal locomotor activity was found between the WT and *Ctsk*^−/−^ mice (Fig. 1B; Day 1: WT = 1031, n = 5, *Ctsk*^−/−^ = 989.5, n = 4; Mann–Whitney U = 10.00, p > 0.999; Day 2: WT = 964, n = 5, *Ctsk*^−/−^ = 1056, n = 4; Mann–Whitney U = 4.00, p = 0.4699; Day 3: WT = 1092.5, n = 5, *Ctsk*^−/−^ = 985.5, n = 4; Mann–Whitney U = 7.00, p = 0.802). However, we cannot rule out that *Ctsk*^−/−^ mice do not have altered responding prior to formalin injection as we did not assess the effect of saline on basal responding prior to formalin injection.

Since formalin-induced pain manifests as more of a transient than chronic inflammatory pain state^31^, complete Freund’s adjuvant (CFA) was used to induce a more chronic inflammatory pain state in WT mice^33,34^. When CFA is injected intraplantar, it causes maximal swelling and inflammation of the paw ~ 24 h after injection and this inflammation remain persistent for 1–2 weeks^35–37^. A key characteristic of this inflammatory pain is hypersensitivity to various mechanical and thermal stimuli^35,36^. Hence, several behavioral paradigms were assayed to measure this hypersensitivity to stimuli. Hypersensitivity to mechanical stimuli causes a decrease in the mechanical paw withdrawal threshold (PWT) and can be termed as mechanical hypersensitivity^38^. In addition, hypersensitivity to thermal stimuli reduces the thermal latency and, therefore can be classified as thermal hypersensitivity^32,38^.

To determine the role of cathepsin K in chronic inflammatory pain, differences in the mechanical PWT between *Ctsk*^−/−^ mice and WT mice after CFA injection were measured. In the first experiment using the traditional method of von Frey testing, a significant attenuation in CFA-induced mechanical hypersensitivity was found in *Ctsk*^−/−^ mice compared to WT mice (Fig. 1C; Day 1: WT = 0.6 g, n = 8, *Ctsk*^−/−^ = 1.4 g, n = 9; Mann–Whitney U = 8.50, p = 0.025; Day 2: WT = 0.8 g, n = 8, *Ctsk*^−/−^ = 1.4 g, n = 9; Mann–Whitney U = 8.00, p = 0.025). This suggests that cathepsin K is necessary for the CFA-induced mechanical hypersensitivity.

Thermal hypersensitivity was assessed in a different cohort of mice at 53 °C in both WT and *Ctsk*^−/−^ mice on day 1 and day 7 post-injection. CFA-injected WT mice had a trend towards a shorter latency to response than the saline-injected WT mice (Fig. 1E; Day 1: Saline = 6.07 s, n = 8, CFA = 5.65 s, n = 7; Mann–Whitney U = 14.00, p = 0.120; Day 7: Saline = 6.61 s, n = 6, CFA = 5.53 s, n = 7; Mann–Whitney U = 6.00, p = 0.068). This tendency was not seen in *Ctsk*^−/−^ mice receiving CFA injection (Fig. 1F; Day 1: Saline = 4.81 s, n = 5, CFA = 5.15 s, n = 5; Mann–Whitney U = 10.00, p = 0.900; Day 7: Saline = 4.79 s, n = 7, CFA = 5.36 s, n = 7; Mann–Whitney U = 21.00, p = 0.900). Of note, the thermal response to saline in *Ctsk*^−/−^ mice was significantly reduced compared to WT (p < 0.001, data not shown). Although we do not believe this changes the interpretation, especially given our pharmacological data, we cannot rule out the possibility of a floor effect and hence a confound in the ability of *Ctsk*^−/−^ mice to adequately remove their paw any faster.

Interestingly, both *Ctsk*^−/−^ and WT mice showed similar amount of inflammatory edema and swelling in the paw on day 7 post-CFA injection after measurement of mechanical hypersensitivity (data not shown). This indicates that cathepsin K does not play a role in inflammation-induced edema but may be altering the transmission of the nociceptive signal.

### Dose–response curve

After seeing no CFA-induced mechanical and thermal hypersensitivity in the *Ctsk*^−/−^ mice, we wanted to see the effect of pharmacological inhibition of cathepsin K activity on the mechanical hypersensitivity in WT mice post-CFA injection. Before proceeding forward, a dose–response correlation study was performed to determine the ideal concentration of the inhibitor.

According to the manufacturer, this inhibitor is specific to cathepsin K, but at higher concentrations it can have off-target effects. For instance, the inhibitor has an inhibition constant of 510 nM for cathepsin B^39^. Therefore, for the dose–response study we chose concentrations less than 510 nM. The tested doses were 0.1 nM, 1 nM, 5 nM, 10 nM, 25 nM, 50 nM, 75 nM, 100 nM, 500 nM, and a vehicle control (0 nM). The data is graphed on a log scale; hence, the vehicle control cannot be seen on the graph.

**Figure 2:**
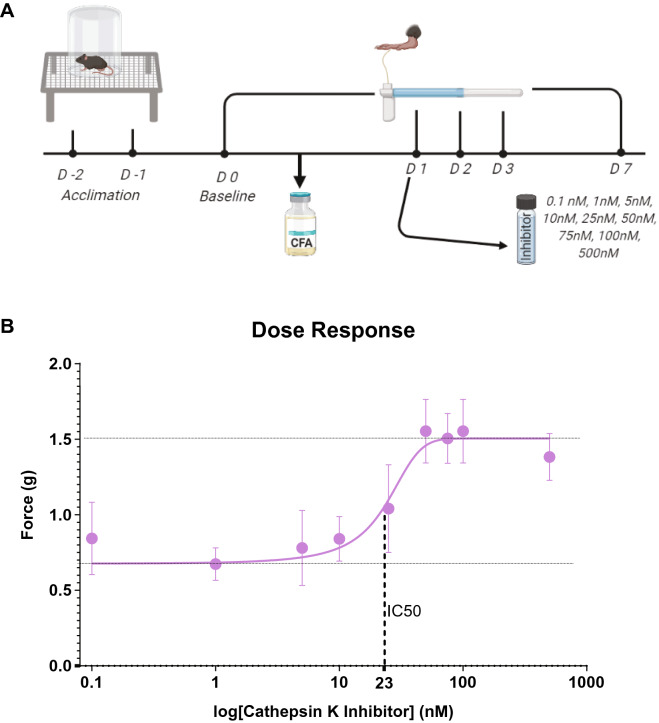
Dose–response curve of cathepsin K inhibitor in mice. (**A**) Timeline for the dose–response curve. Nine different concentrations of cathepsin K inhibitor were selected. Each group of WT mice was given an injection of one of the nine different concentrations of the cathepsin K inhibitor on day 1 post-CFA (complete Freund’s adjuvant) injection. Their response to each concentration of the inhibitor in terms of force (g) needed to induce paw withdrawal was recorded and these responses were used to plot a dose–response curve. (**B**) The calculated IC50 of the inhibitor is 23.26 nM, the top of the response curve lies at 1.51 g and the bottom of the response curve lies at 0.61 g. Of the doses tested, the inhibitor starts to show an effect at 5 nM and the effect peaks at 50 nM. Data are represented as mean ± SEM.

On day 1, following CFA injection, but before testing the PWT all mice received the inhibitor injection at the same site as that of CFA injection the day before. The observed PWT for each concentration was used to plot a dose response curve (Fig. 2B). According to this curve the extrapolated IC50 of the inhibitor is at 23.26 nM. The PWT for the top of the response curve lies at 1.5 g and the bottom of the response curve lies at 0.6 g. Of the doses tested, the inhibitor shows no effect at 1 nM or less, whereas the effect peaked at 50 nM. Therefore, our future studies that expounded upon the effectiveness of the inhibitor used ~ twice the IC50 (50 nM). The 50 nM concentration is also the concentration at which the effect of the inhibitor peaks. Increasing the dose further could potentially raise the chances for the side-effects of the drug, if there would be any, without having any additional beneficial effects.

### Cathepsin K inhibitor-injected mice (50 nM) do not display mechanical hypersensitivity

**Figure 3:**
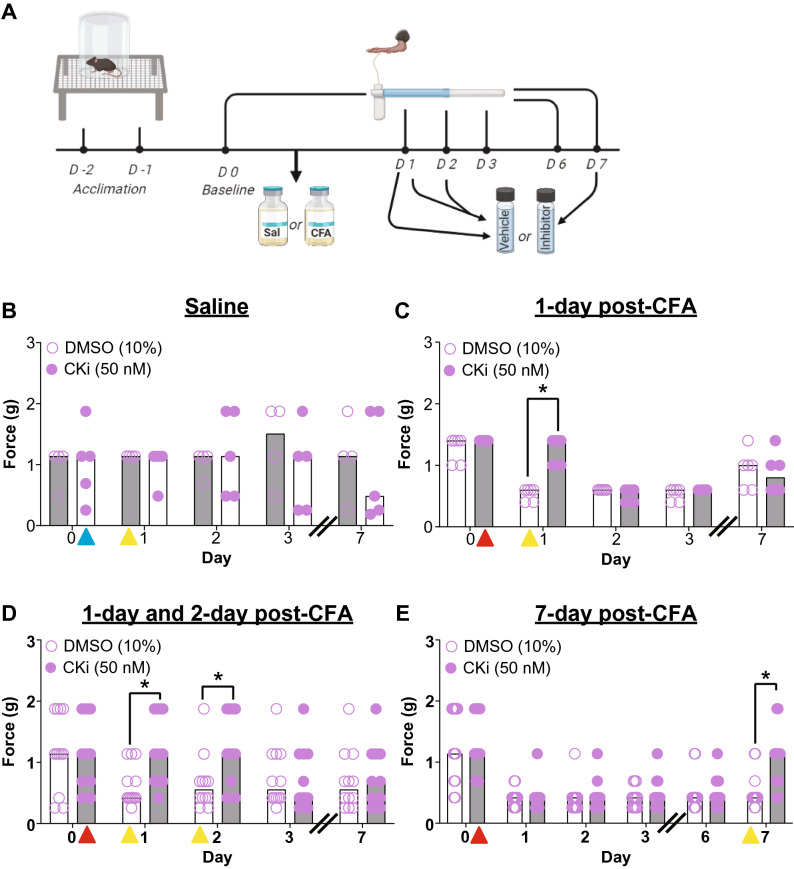
Cathepsin K inhibitor (50 nM) reduces mechanical hypersensitivity in mice. (**A**) Timeline for testing mechanical hypersensitivity. (**B**) There is no effect of the 50 nM cKi when given in mice with saline injection. Blue and yellow triangle indicates the time of saline and cKi injection respectively. (**C**) Whereas a single injection of 50 nM cKi (yellow triangle) when given before testing on day 1 post-CFA (complete Freund’s adjuvant) injection (red triangle) results in higher mechanical threshold as compared to vehicle (10% DMSO in saline) injection. (**D**) During the developmental phase of chronic inflammatory pain (i.e., on day 1 and day 2 post-CFA injection), subsequent injections of 50 nM cKi can alleviate hypersensitivity compared to vehicle injection. (**E**) Similarly, during the maintenance phase of persistent inflammatory pain (i.e., on day 7 post-CFA injection) 50 nM cKi injection reduces mechanical hypersensitivity when compared to vehicle injection. Data are represented at scatter plots of individual mice with bar graphs overlayed indicating the median value. *p < 0.05 Mann–Whitney test between cKi and vehicle treatment for each day.

Since the traditional method for detecting differences in von Frey sensitivity can result in mice receiving a varying number of von Frey filaments, it can lead to a different testing experience in each mouse^40^. On the contrary, in SUDO method each mouse receives the same number of von Frey filaments, and therefore, moving forward, the SUDO method was used for all mechanical hypersensitivity experiments, except in Fig. 3C, in which the traditional method was used.

Before establishing whether there was a therapeutic effect of cKi (50 nM) we ruled out the possibility that there was an off-target effect of the cKi on the basal mechano-sensitivity (Fig. 3B, Day 1: DMSO(10%) = 1.13 g, n = 4, cKi(50 nM) = 1.13 g, n = 5; Mann–Whitney U = 8.00, p > 0.999; Day 2: DMSO(10%) = 1.13 g, n = 4, cKi(50 nM) = 1.13 g, n = 5; Mann–Whitney U = 9.50, p > 0.999; Day 3; DMSO(10%) = 1.50 g, n = 4, cKi(50 nM) = 1.13, n = 5; Mann–Whitney U = 6.00, p = 0.909; Day 7; DMSO(10%) = 1.13 g, n = 4, cKi(50 nM) = 0.48, n = 5; Mann–Whitney U = 8.5, p = 0.997). Hence, we recapitulated what we observed with the dose–response experiment and presumably established a therapeutic effect with 50 nM cKi. The effect of the inhibitor was transient, that is 1 day post-CFA injection, there was a significant difference in the PWT between vehicle- and cKi-injected mice (Fig. 3C; DMSO (10%) = 0.6 g, n = 6; cKi(50 nM) = 1.4 g, n = 6; Mann–Whitney U = 0.00, p = 0.010). Yet, on day 2 post-CFA injection following cKi treatment (with no inhibitor onboard) there was no longer an observable effect (Fig. 3C; DMSO (10%) = 0.6 g, n = 6; cKi(50 nM) = 0.6 g, n = 6; Mann–Whitney U = 12.00, p = 0.911). This suggests that the drug was either metabolized or washed out from the system in less than a day and that cathepsin K activity is necessary for the expression of acute mechanical hypersensitivity but does not necessarily prevent the persistent expression of mechanical hypersensitivity when cathepsin K activity returns.

Using the inhibitor, we attempted to parse out the role of cathepsin K in different phases of chronic inflammatory pain. Hence, to test the effect of cathepsin K inhibition during the establishment phase of chronic pain, the cKi was given before mechanical PWT testing on both days 1 and 2 post-CFA injection. Consistent with our previous experiment (Fig. 3C) cKi-injected mice had a significantly higher PWT on day 1 (Fig. 3D, DMSO (10%) = 0.41 g, n = 12; cKi(50 nM) = 1.13, n = 13; Mann–Whitney U = 26.00, p = 0.017) which was maintained on day 2 (DMSO (10%) = 0.55 g, n = 12; cKi(50 nM) = 1.13 g, n = 13; Mann–Whitney U = 30.00, p = 0.029). However, on day 3, when no cKi was delivered the mechanical hypersensitivity returned to control levels (DMSO (10%) = 0.55 g, n = 12; cKi(50 nM) = 0.41 g, n = 13; Mann–Whitney U = 66.00, p = 0.897). Suggesting the analgesic effect is only transient while the drug is on board.

To further test whether cathepsin K inhibition could produce analgesia during the maintenance phase of persistent pain the cKi was injected 7 days after CFA injection. The cKi-injected mice had significantly higher PWT compared to vehicle-injected controls on day 7, when they were injected with the cKi (Fig. 3E; DMSO (10%) = 0.41 g, n = 13; cKi(50 nM) = 1.13 g, n = 13; Mann–Whitney U = 27.50, p = 0.011). Based upon our data, we conclude that inhibition of cathepsin K activity is therapeutically viable to produce analgesia during both the establishment and maintenance phases of chronic inflammatory pain. However, the effect is transient and does not appear to alter the progression of the disease state.

### Cathepsin K inhibitor-injected (50 nM) mice have attenuated thermal hypersensitivity

**Figure 4:**
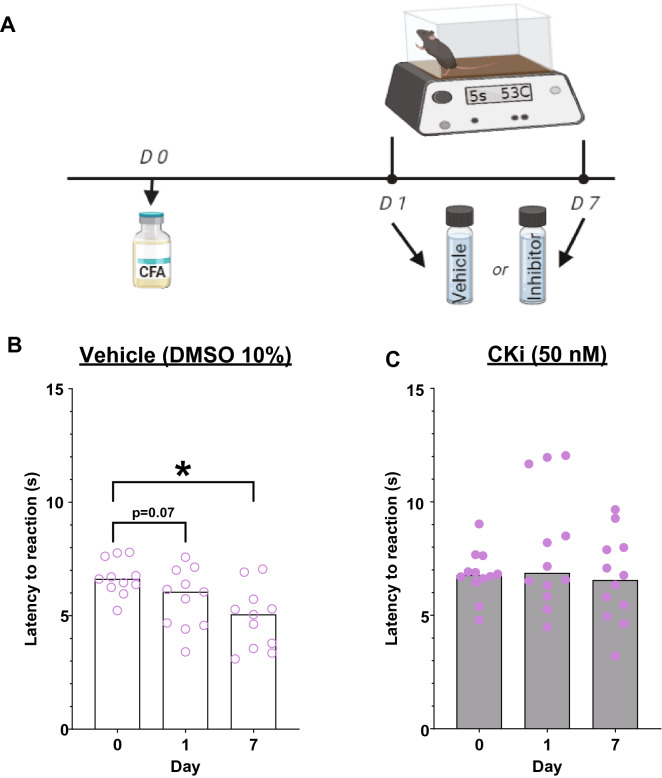
Cathepsin K inhibitor (50 nM) reduces thermal hypersensitivity in mice. (**A**) Timeline for testing thermal hypersensitivity. (**B**) Vehicle-injected mice have a significantly lower thermal latency than day 0 (baseline) on day 7 post-CFA injection. (**C**) 50 nM cKi-injected mice show no significant change in thermal latency than day 0 (baseline) post-CFA injection. Data are represented at scatter plots of individual mice with bar graphs overlayed indicating the median value. *p < 0.05 Mann–Whitney test between time points for each treatment.

We also assessed the effect of cKi on thermal hypersensitivity. For this, cKi was injected in the same animals (counterbalanced) prior to testing on day 1 and day 7 after the CFA injection. Mann Whitney test showed that the vehicle-injected mice had a significant decrease in thermal latency from baseline on day 7, post-CFA (Fig. 4B; Day 0 = 6.62 s, n = 11; day 7 = 5.05 s, n = 11; Mann–Whitney U = 19.00, p = 0.005) and strong trend on day 1, post-CFA (Fig. 4B; Day 0 = 6.62 s, n = 11; day 1 = 6.05 s, n = 11; Mann–Whitney U = 33.00, p = 0.075) but there was no change in the thermal latency from baseline in the cKi-injected mice post-CFA injection (Fig. 4C, Day 0 = 6.76 s, n = 12; Day 1 = 6.86 s, n = 12; Mann–Whitney U = 66.00, p = 0.755; and Day 0: = 6.76 s, n = 12; day 7 = 6.55 s, n = 12; Mann–Whitney U = 68.00, p = 0.842) and the thermal latency of cKi-injected mice was similar to day 0 thermal latency of vehicle-injected mice.

As in the *Ctsk*^−/−^ mice, pharmacological inhibition of cathepsin K activity also does not change the amount of inflammatory swelling or edema, as the paw diameters and weights were similar in inhibitor and vehicle-injected mice after the completion of hot plate test on day 7 post-CFA injection (data not shown). This shows that the inhibitor is not reducing the collective inflammatory edema and infiltration, is significantly reducing the inflammatory pain (expression and/or transmission).

### Cathepsin K mRNA, protein and activity

We compared the cathepsin K mRNA, protein, and protein activity levels between CFA-injected mice and saline-injected mice on day 1 and day 7 post-CFA injection. Our biochemical analysis demonstrates that cathepsin K mRNA, protein and activity levels increase in the hind paw of WT mice 1 day and 7 days after CFA injection into the hind paw.

**Figure 5:**
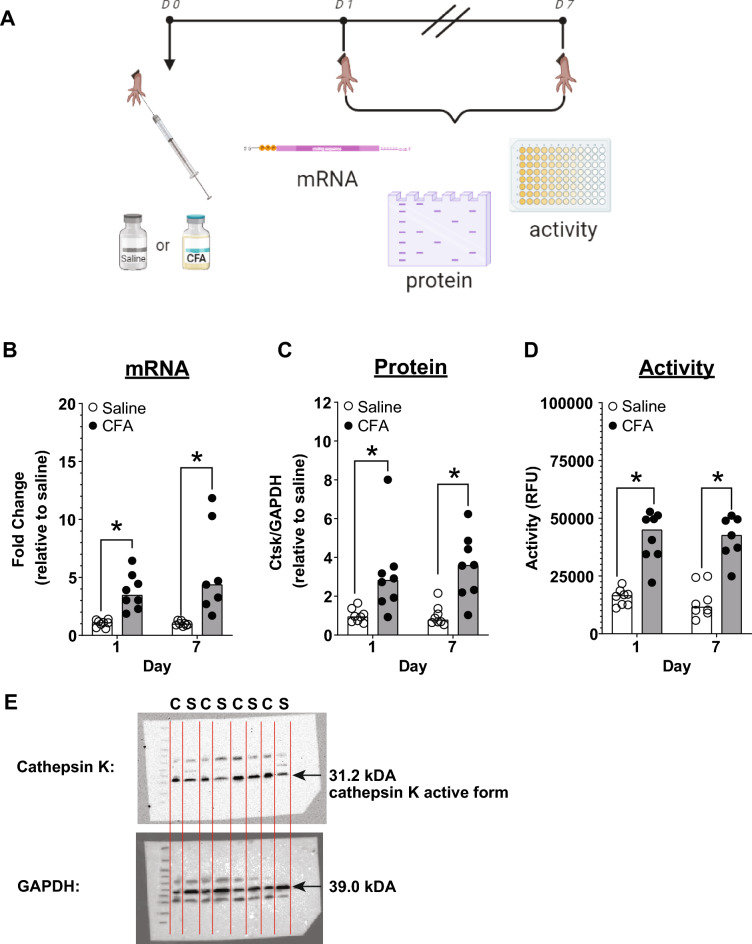
Cathepsin K levels rise post-inflammation in mice. (**A**) Timeline for testing mRNA, protein levels and protein activity. (**B**) There is an increase in cathepsin K mRNA levels in the hind paw after CFA injection. (**C**) Increased cathepsin K protein (active-form) in hind paw after CFA injection. (**D**) In addition, there is an increase in cathepsin K activity in the hind paw after CFA injection. (**E**) Representative western blot of cathepsin K and housekeeping protein GAPDH after CFA injection. Black arrow indicates the molecular weight of detected protein. CFA and Saline treated samples are represented as C and S, respectively. Data are represented at scatter plots of individual mice with bar graphs overlayed indicating the median value. *p < 0.05 Mann–Whitney test between saline and CFA treatment on day 1 and day 7.

Cathepsin K mRNA levels are higher in the paw of CFA-injected mice (n = 8) compared to saline-injected mice (Fig. 5B; Day 1: Saline = 1.10, n = 8; CFA = 3.47, n = 8; Mann–Whitney U = 0.00, p = 0.0003; and Day 7: Saline = 0.97, n = 8; CFA = 4.37, n = 7 Mann–Whitney U = 12.00, p = 0.0003). Moreover, our Western blots showed a significant increase of cathepsin K active-form compared to saline- injected mice on day 1 and 7 post-CFA injection (Fig. 5C; Day 1: Saline = 0.94, n = 8; CFA = 2.82, n = 8; Mann–Whitney U = 5.00, p = 0.002; and Day 7: = 0.77, n = 8; CFA = 2.82, n = 8; Mann–Whitney U = 3.00, p = 0.002), and post-CFA injection there is also an enhancement in cathepsin K activity on day 1 and day 7 post-CFA injection compared to saline controls (Fig. 5D; Day 1: Saline = 16532RFU, n = 8; CFA = 44992RFU, n = 8; Mann–Whitney U = 0.00, p = 0.0003; and Day 7: = 11562RFU, n = 8; CFA = 42592RFU, n = 7; Mann–Whitney U = 1.00, p = 0.0006. This data suggests that cathepsin K levels are increasing after the CFA insult.

## Discussion

This study reports a potential role for cathepsin K in cutaneous persistent inflammatory pain. Our studies suggest that cathepsin K mRNA, protein, and activity go up in mice with CFA-induced inflammation (Fig. 5). In addition, when cathepsin K is low or absent, like in *Ctsk*^−/−^ mice (Fig. 1) or in mice with pharmacological inhibition of cathepsin K (Figs. 2, 3, 4), the CFA-induced persistent inflammatory pain is attenuated. In addition to the CFA-induced mechanical and thermal hypersensitivity, the lack of nocifensive behaviors observed in the *Ctsk*^−/−^ mice in the late phase of formalin test further strengthens our hypothesis that cathepsin K mediates inflammatory pain as nocifensive behaviors seen in the late phase are thought to be due to inflammatory responses in the peripheral tissue and functional changes in the spinal dorsal horn^31^.

In our models the cellular sources of cathepsin K at the site of inflammation has not yet been determined. Cathepsin K is abundant in osteoclasts^41^ but because of the superficial location of the inflammatory insult in our models and fast onset of symptoms, especially in the case of the formalin injection, it is unlikely that most of cathepsin K activity is coming from the deeply seated osteoclasts. Cathepsin K is also known to be associated with other cells like epithelial cells, macrophages, dendritic cells, fibroblasts, neurons, and glia^42–46^ and because of their superficial location in the paw any of these can be a potential primary source of cathepsin K at the site of inflammation in our models. Notably, macrophages contribute to the extracellular release in our models as osteoclasts are a macrophage derivative and cathepsin K is a macrophage maturation marker^43^. Inside the cells, cathepsin K is usually present in the lysosomes, which have an acidic pH. Under physiological conditions if cathepsin K would be released in the extracellular matrix it more than likely would be quickly destroyed at the neutral pH. However, inflammation lowers the pH^47^ and may allow for activation and stabilization of cathepsin K, which could permit increased and sustained activity of cathepsin K at the site of the inflammatory insult. Thus, inflammation may provide the necessary microenvironment for cathepsin K to play a critical role in pain signaling.

Although the mechanism of action by which cathepsin K is working to mediate mechanical and thermal hypersensitivity is beyond the scope of this paper; we speculate that cathepsin K, like cathepsin S might be causing nociception through the cleavage of PAR2 on nociceptors^18,48^. We favor this hypothesis because cathepsin K is similar in structure to cathepsin S^49^ and cathepsin K has been shown in tissue where PAR2 is expressed^50^. It is worth pointing out that due to the similarity in structure of cathepsins K, S, and V, we cannot rule out that the activity assay solely detected cathepsin K activity as the substrate is cleaved by several cysteine cathepsins^51^. However, this data in conjunction with changes in cathepsin K mRNA (Fig. 5B) and protein expression (Fig. 5C) indicate cathepsin K levels are altered with CFA injection. In addition, the effectiveness of our genetic deletion studies using Ctsk^−/−^ mice (Fig. 1), and pharmacological studies (Figs. 2, 3, 4) strongly suggest cathepsin K plays a role in cutaneous inflammatory pain.

Cathepsins, are known to take part in extracellular matrix remodeling^16,17,52^. Cathepsin K, S, and V have been shown to play a role in cardiovascular diseases by causing excessive elastin degradation^16^. Involvement of cathepsin B and L has been shown in neural tissue remodeling^16^. Activity-dependent exocytosis of cathepsin B from lysosomes has been shown to alter structural plasticity of dendritic spines and cause extracellular matrix remodeling in the hippocampal pyramidal neurons^53^. Axons of the cerebellar granule cells have also been shown to release cathepsin B in an activity-dependent manner, which initiated synaptogenesis^54^. Since cathepsin K is well known for breaking down extracellular matrix and is a key player in bone remodeling^28,55,56^ it is possible that cathepsin K might be causing similar changes in the ascending pain pathways, which could augment the peripheral inflammatory responses and further increase the chemically induced hypersensitivity.

Overall, our data aligns with the currently available literature on cathepsins. Like cathepsin B knockout mice, *Ctsk*^−/−^ mice, do not develop mechanical hypersensitivity after CFA injection and the inflammatory infiltrates and paw diameters are also similar in cathepsin B knockout and WT mice after CFA injection^23^. Similarly, there was also an absence of mechanical hyperalgesia in cathepsin E knockout mice in an experimental model for autoimmune encephalitis^25^ and in cathepsin S knockout mice in a neuropathic pain model^21^.

Like the cathepsin K inhibitor used for these studies, EMD 219379, the cathepsin G inhibitor TPCK also reduces the thermal hypersensitivity associated with CFA-induced inflammatory pain^27^. Similarly, cathepsin S inhibitors also reduces mechanical hypersensitivity in mice with neuropathic pain^19^ and provoked pain in mice with formalin injection^18^. Previously, another cathepsin K inhibitor L-006235 was reported to have a similar effect in MIA- (monosodium iodoacetate-) induced model of painful osteoarthritis. Animals receiving L-006235 post-arthritis induction displayed less pain and there was no effect on the inflammation histologically. Therefore, it was suggested that L-006235 only reduced the pain signaling^57^. Finally, inhibition of cathepsin K with NC-2300, a potent orally active cathepsin K inhibitor was able to ameliorate CFA-induced paw swelling and improve locomotive activity suggesting cathepsin K participates in autoimmune inflammation^58^. Further studies are needed to determine why paw swelling was reduced with the orally active cathepsin K inhibitor, which we did not observe in *Ctsk*^−/−^ mice or local inhibition within the hind paw.

However, in contrast to our study where acute injection of the inhibitor reduced nociceptive signaling, Nwosu et al.^59^ showed that inhibition of cathepsin K activity reduced nociception only after long-term treatment. The onset was said to be slow because of effects on pain mechanisms other than acute nociception^57^. Likewise, another cathepsin K inhibitor AZ12606133 also reduced the firing rate in osteoarthritic joint’s mechanosensory afferents but only 28 days after the onset of intrathecal administration and not 15 min after intra-arterial injection^30^. A possible explanation for why our treatment works acutely may be that in the arthritic joints, most probably the osteoclasts secrete the vast majority of cathepsin K. Therefore, initially most of the inhibitor molecules are bound to and utilized by the osteoclastic cathepsin K. Once the osteoclastic cathepsin K lowers the inhibitor can be more evenly distributed and more inhibitor molecules are available for binding to the cathepsin K in the other cell types that could be involved in producing the hypersensitivity. The fast onset seen in our study could also be due to fact that in our study we locally inhibited cathepsin K at the site of the inflammatory insult, rather than systemically or in the CNS. This is also important since Odanacatib, another cathepsin K inhibitor was withdrawn in phase 3 of clinical trials because it increased the number of adverse cardiovascular events and atypical fractures^60^. Odanacatib was being prescribed orally. Local inhibition by EMD 21379 will greatly reduce these side-effects if these adverse side-effects might be associated with this molecule as well. However, EMD 21379 does not have the same selectivity as Odanacatib and we cannot rule out the contribution of partially inhibiting cathepsin B, although minor (Ki apparent = 510 nM), and/or papain (Ki apparent = 1.2 µM) has on the inflammatory response^23^. In addition, whether EMD 21379 may inhibit cathepsin S and V has not been reported, which are both expressed in the epidermis^61^. Nevertheless, the pharmacological studies combined with the lack of a response in *Ctsk*^−/−^ mice and the increases in cathepsin K mRNA, protein, and activity strongly suggest cathepsin K plays a role in inflammatory-induced nociception.

As seen above, some cathepsins like cathepsin S are known to be involved in neuropathic pain^19^ and differential expression of cathepsin S and X in the spinal cord of neuropathic pain models in rats has also been reported^20^. Therefore, future studies will see if cathepsin K also plays a role in neuropathic pain. Our data also suggest that cathepsin K could be involved in neuropathic pain, as *Ctsk*^−/−^ mice showed almost no nociceptive behavior in the late phase of the formalin test and drugs that have been effective at attenuating the late phase have preclinically been shown to be effective at reducing neuropathic pain^62–64^.

CFA-induced persistent inflammatory pain models in rodents also closely mimics the time course of persistent injury in humans^32^. Since inhibition of cathepsin K activity after 7 days of persistent nociception was able to reverse the CFA-induced mechanical hypersensitivity this suggests that cathepsin K inhibitors may be used after the establishment of pain and provide a novel treatment strategy (Fig. 3E). Hence, although our data suggest that pharmacological inhibition reduces chronic inflammatory hypersensitivity (Figs. 3) further research is needed to determine the parameters by which inhibition of cathepsin K activity produces analgesia and whether attenuating cathepsin K activity may generate a clinically relevant therapeutic as the transient nature of the inhibition still needs to be parsed out. We do recognize that females tend to be more sensitive to nociceptive stimuli and outnumber males as suffers of persistent pain^65^ and based on sex, difference in GABA signaling in the descending pain modulatory circuit has been shown post CFA-induced persistent inflammation^66^. Hence, studies in female animals are essential and our future studies will address it. Regardless, our studies have laid the framework for future studies to determine the viability of targeting cathepsin K activity to reduce inflammatory-mediated pain.

## Methods

This study is reported in accordance with ARRIVE guidelines. Number of animals used in each experiment is listed in Table 1.

**Table 1:** Number of mice used per experiment.

	Experiment	WT	Ctsk^−/−^
Figure 1A	Formalin Injection—Nocifensive behavior	8	8
Figure 1B	Locomotor activity	5	4
Figure 1C,D	Mechanical Hypersensitivity (Traditional Method)	8	9
Figure 1E,F	Thermal Hypersensitivity	28	24
Figure 2B	Dose Response curve (Cathepsin K inhibitor)	54	
Figure 3	Mechanical Hypersensitivity following Cathepsin K inhibitor treatment	72	
Figure 4	Thermal Hypersensitivity following Cathespin K inhibitor treatment	17	
Figure 5A	mRNA expression following CFA injection	31	
Figure 5B	Cathepsin K activity following CFA injection	31	
	Total mice	254	45

### Animals

All procedures were performed in accordance with the National Institutes of Health’s *Guidelines for the Care and Use of Laboratory Animals* and with approval from the Institutional Animal Care and Use Committee at the University of Wyoming. A total of 254 male C57BL/6 mice (WT) and 45 male *Ctsk*^−/−^ mice^29^ were used for these experiments. All mice were bred in-house and kept in a temperature-controlled room with a 12-h light/dark cycle and had free access to food and water and were age-matched between 8 and 16 weeks old during the experiment. Animals were socially housed in transparent boxes with bedding made of softwood shavings. All efforts were made to minimize the number of animals used in the experiments and to reduce the amount of pain and suffering. The number of mice per group for each experiment was determined a priori and is based on the statistical power needed to reliably detect the group differences given the measures to be made, the expected variance and size effect.

### Behavioral analysis

All behavioral experiments were performed during the light cycle. Mice used for behavioral analysis were handled/transported in cups to minimize the stress. They were also acclimatized to the experimental apparatus before the start of each study for a minimum of 1 h, at least twice prior to the initiation of the experiment. For mechanical hypersensitivity testing, mice were also acclimatized for an hour on each testing day. For studies spanning several days, testing was performed around the same time each day and was run in counterbalanced manner. In studies using different genotypes, age-matched mice were used. In studies using the same genotype, littermates were used as controls. For peripheral inflammation and inflammatory pain, either complete Freund’s adjuvant [CFA (Sigma#F5881), 1:1 v/v CFA to saline] or 5% formalin (Fisher Scientific#R31750004A) was injected into the right hind paw subcutaneously into the plantar surface. Saline (0.9% Sodium Chloride, Hospira#RL-4494) was injected as a control to account for possible mechanical trauma of the injection. In pharmacological studies, WT mice were injected with either 10 μL of cathepsin K inhibitor II (EMD Millipore#21937, 1-(*N*-benzyloxycarbonyl-leucyl)-5-(*N*-Boc-phenylalanyl-leucyl)carbohydrazide)^39^ or its vehicle (10% DMSO in saline) 1–2 h before testing. Cathepsin K inhibitor or vehicle injections were given locally (at the same site of CFA-induced inflammation).

### Mechanical hypersensitivity

The minimal force in grams (g) needed to display an adequate pain response is termed as the mechanical paw withdrawal threshold (PWT). Mechanical PWT was measured to assess mechanical hypersensitivity after a chemical insult. To test the PWT, von Frey filaments (North Coast Medical) ranging from 0.16 to 4.0 g were used. The filaments were either presented in ascending order until the animal displayed a nocifensive response (traditional method^67^) or in a simplified up-down fashion (SUDO method^40^). In the traditional method, von Frey filaments are applied in an ascending order starting from the bottom of the force range until the mouse displays a nocifensive response to more than five out of ten applications^67^. Whereas in the SUDO method, von Frey filaments are applied starting from the middle of the force range. Depending on whether a response is seen or not seen in five out ten applications of a filament, the next filament applied is of a lower or a higher force. In this manner a total of five filaments are applied in SUDO method^40^. Figure 3A shows the experimental timeline for studies looking at the effect of the cathepsin K inhibitor (cKi) on the mechanical PWT. Following the initial baseline testing in the right hind paw, mice received 12 μL of CFA or saline only into the right hind paw. Testing was then repeated on days 1, 2, 3, 7, and if need be, on day 6 post-injection. The experiments measuring the difference in mechanical hypersensitivity in WT and *Ctsk*^−/−^ mice were performed by two independent experimenters using the two different methods described above. All pharmacological experiments, except the one shown in Fig. 3C were performed using the SUDO method.

### Thermal hypersensitivity

The latency to a nocifensive response (seconds), when placed on a hot plate (IITC Life Science Inc.) at 53 °C, was measured and termed as the thermal latency. Transient Receptor Potential (TRP) channels are thermosensors in sensory nerves and since downstream TRP channel associated with cathepsin K is not known and all 4 TRP heat sensing channels are active at 53 °C, we choose to measure thermal latency at 53 °C^68^. This thermal latency was used to assess thermal hypersensitivity after a chemical insult. Each mouse was tested three times at an interval of 20 min. The average time to response was noted^69^. The mouse was taken off the hot plate immediately after a nocifensive response was observed. Initial experiments between WT and *Ctsk*^−/−^ mice were performed by two different experimenters using the same method. The final data set shows the combined results. The inhibitor studies were performed by a single experimenter. Figure 4A shows the experimental timeline for the studies looking at the effect of the cathepsin K inhibitor (cKi) on the thermal hypersensitivity.

### Formalin test

WT and *Ctsk*^−/−^ mice were habituated in the experimental chamber after which they received 20 μL of 5% formalin into the right hind paw. Immediately after the injection, the mice were put back in the chamber and were video recorded for 40 min. Formalin injection induced an unprovoked display of nocifensive behaviors such as licking, biting, and flinching of the paw. The total time spent displaying these responses during each 5-min window was noted^70^. Experiments were performed by a single experimenter, but the recordings were analyzed by a second person who was blinded to the genotype.

### Paw diameter and paw weight measurements

Change in paw diameter and weight due to swelling and edema was measured as a parameter of paw inflammation. Paw diameter was measured at the site of maximal swelling using a standard caliper, and paw weight was measured using a standard weighing scale after the removal of the paw at the hairline following the completion of the experimental study.

### Locomotor activity

WT and CK^−/−^ mice were acclimatized to the experimental apparatus for 5 min. After acclimation, beam breaks were measured for 15 min in one side of a three chambered conditioned place preference box (Med Associates). The experiment was performed similarly for 3 consecutive days. The total number of beam breaks on each day was used to determine the horizontal locomotor activity.

### Quantitative RT-PCR

Paws were isolated from saline- or CFA-injected WT mice after 1 day or 7 days and cut into small pieces. Then the tissue was homogenized, lysed in Trizol reagent (Invitrogen#1559606), and the RNA was extracted for qRT-PCR. Briefly, 1 mL Trizol was added to cut tissues and homogenized using Dounce homogenizer. Next, chloroform (0.2 mL) was added to Trizol cell lysate, mixed, and incubated at room temperature for 5 min. The samples were then centrifuged at 12,000*g* for 15 min. The aqueous phase was shifted to a fresh tube. Isopropyl alcohol (1 mL) was added to it and incubated for 10 min, and then the sample was centrifuged at 12,000*g* for 10 min. The supernatant was discarded, and the RNA pellet was washed in 70% ethanol and centrifuged at 12,000*g* for 5 min. The supernatant was discarded, and the pellet was dried in air to remove any ethanol. The RNA pellet was dissolved in 50 μL of RNAase-free dH2O. The RNA was quantified by using a Nanodrop (Thermo Scientific).

The isolated RNA was transcribed into cDNA using RT2 First Stand Kit (Qiagen, 330404). Briefly, RNA (5 μg) was incubated with genomic DNA Elimination Buffer for 5 min at 42 °C in a Minicycler (Bio-Rad). RT Cocktail (RT buffer, Primers, RT enzyme mix and RNase-free H20) was added to DNA Elimination mixture and incubated at 42 °C for 15 min. The reaction was stopped immediately by heating at 95 °C for 5 min and dH20 (91 μL) was added to each cDNA synthesis reaction. Cathepsin K expression was determined by qRT-PCR by mixing synthesized cDNA, cathepsin K primers (5′-GGG CCA GGA TGA AAG TTG TA-3′, 5′-CAC TGC TCT CTT CAG GGC TT-3′), and SsoAdvanced Universal SYBR supermix (Biorad, 1725270) and placed in CFX connect (Biorad). The samples were heated at 95 °C for 10 min, and then 40 cycles were run at: 95 °C for 15 min, 55 °C for 40 s, and 72 °C for 30 s. Expression of housekeeping gene *GAPDH* (primer-qiagen#249900) was determined to normalize gene expression. The data was collected using Bio-Rad CFX manager and relative cathepsin K fold change is calculated using 2 ^(−ΔΔCT)^ method between CFA and Saline groups.

### Western blotting

Paw was isolated from saline or CFA injected mice and cut into small pieces. Then the tissues were homogenized in RIPA buffer containing protease inhibitor cocktail (Thermo Scientific, Waltham, MA) using dounce homogenizer. Protein concentration in the lysates was measured using Nanodrop (Thermo). Protein samples (40 µg) were then mixed with DTT containing Laemmli’s buffer and incubated at 99 °C for 10 min and were separated via Polyacrylamide Gel Electrophoresis (PAGE) using 4–15 gradient gel. After the separation, protein samples were transferred to PVDF membrane and probed using primary antibodies against cathepsin K mouse anti-cathepsin K (SC-48353, 1:500) and Gapdh (ab9483, 1:1000). The protein bands were visualized using freshly prepared Super-Signal West Femto Kit (Pierce) and Gel Doc XRS digital imaging system (Bio-Rad) was used to capture images. Once the images were captured, Restore PLUS western blot stripping buffer (Thermo Scientific) was used to strip the membrane before reprobing. Densitometry analysis was carried out using Quantity One software (Biorad). Cathepsin K protein was normalized to housekeeping protein GAPDH. Blots were compared by internally normalizing the blot to corresponding WT control (saline) group.

### Activity assay

Cathepsin K activity was measured using cathepsin K Activity Assay Kit (BioVision, K141-100) following the manufacturer’s instructions. Figure 5A shows the experimental timeline for the studies evaluating changes in cathepsin K levels. Paws were removed from saline- or CFA-injected WT mice after 1 or 7 days. After removal, the paws were cut into small pieces and processed following the manufacturer’s instructions. Briefly, cut tissue was lysed using cell lysis buffer for 10 min on ice and supernatant was collected. Protein concertation of the supernatant was determined using Pierce BCA protein Assay Kit (Thermo Scientific, 23225). Equal amount of isolated protein was mixed with reaction buffer and substrate 200 µM Acetyl-Leu-Arg-7-amido-4(trifluoromethyl)-coumarin (Ac-LR-AFC). The reaction mixtures were incubated at 37 °C for 90 min and examined using a fluorometer at 400-nm excitation and 500-nm emission. Fold-increase in cathepsin K activity in CFA-injected mice was determined by comparing the relative fluorescence units with the levels of saline-injected mice.

### Data analysis

The responses in many of the data sets in this study involved counts which could be small or these responses involved ties where the values could be duplicated. As a result, there were legitimate concerns with the assumptions of normality that would be required of parametric analyses. Given these suspected violations of the assumptions, all analyses were conducted using a nonparametric approach^71^. The remaining analyses for responses without these concerns were also conducted using a nonparametric approach for consistency. The nonparametric approach was more conservative than the parametric approach in this study as it confirmed the statistically significant differences for the parametric approach in all comparisons, except in a few cases.

With the nonparametric approach, multiple comparisons were performed to evaluate group differences across each of the time points using the Holm–Sidak adjustment, which is a refined version of the Bonferroni adjustment (GraphPad Prism, 2022). While this approach adjusts for the multiple tests, it assumes equal variances across the comparisons, which is in accordance with the assumption of sphericity^72^. Nevertheless, this approach is moderately robust to small deviations from this assumption of sphericity as it is a refinement of the Bonference adjustment that has been recommended in such circumstances^72^. Multiple Mann–Whitney tests were used for formalin, locomotor activity, mechanical hypersensitivity, cKi hot plate, mRNA, protein, and activity analysis experiments using GraphPad Prism software (Version 9.1.2, San Diego, CA). Mann–Whitney test was used for WT vs. KO hot plate experiments using the same GraphPad Prism software. A p value of < 0.05 is considered statistically significant. There were no exclusions of any animal or data points.

## Data Availability

The datasets used and/or analyzed during the current study available from the corresponding author upon reasonable request.
